# Correlations among Pulmonary DJ-1, VDR and Nrf-2 in patients with Chronic Obstructive Pulmonary Disease: A Case-control Study

**DOI:** 10.7150/ijms.58452

**Published:** 2021-04-22

**Authors:** Ying Xiang, Lin Fu, Hui-Xian Xiang, Ling Zheng, Zhu-Xia Tan, Li-Xiang Wang, Wei Cao, De-Xiang Xu, Hui Zhao

**Affiliations:** 1 Respiratory and critical care medicine, Second Affiliated Hospital of Anhui Medical University, Hefei, 230601, China.; 2 Department of Toxicology, Anhui Medical University, Hefei, 230032, China.

**Keywords:** DJ-1, VDR, Nrf-2, lung, COPD, pulmonary function

## Abstract

Parkinson protein 7 (PARK7)/DJ-1 (DJ-1) is a redox sensitive molecular and stabilizer of nuclear factor erythroid 2-related factor 2 (Nrf-2). Nrf-2 regulates the downstream antioxidant defense system and exerts a significant function in patients with chronic obstructive pulmonary disease (COPD). Vitamin D receptor (VDR) is the nuclear receptor that regulates the downstream target genes. This study aimed to analyze the associations among pulmonary function, DJ-1, VDR and Nrf-2 in COPD patients. Serum was collected from 180 COPD patients and control subjects. Thirty-five lung tissues were obtained. DJ-1 was measured using ELISA and western blotting. Nrf-2 and VDR were detected by immunohistochemistry. Serum and pulmonary DJ-1 levels were lower in COPD patients than those in control subjects. Pulmonary VDR-positive nuclei were reduced in COPD patients. Nrf-2-positive nuclei were reduced in lung tissues of COPD patients. On the contrary, Nrf-2-related downstream target proteins were elevated in COPD patients. Further correlation analysis indicated that forced expiratory volume in 1 second (FEV1) was positively associated with pulmonary DJ-1, VDR and Nrf-2 in patients with COPD. In addition, there were positive correlations among DJ-1, VDR and Nrf-2 in lung tissues of COPD patients. In conclusion, DJ-1, VDR and Nrf-2 were decreased in COPD patients compared with control subjects. The reduction of DJ-1 and VDR associating with Nrf-2 downregulation may be involved in the process of COPD.

## Introduction

Chronic obstructive pulmonary disease (COPD), which accompanied with irreversible airflow limitation and chronic airway inflammation, is the third dominating reason of morbidity and mortality and result in tremendous economic pressures of health care all over the world 1. The pathogenesis and pathology of COPD are complex, but excessive airway inflammation reaction, oxidative stress, imbalance between protease/antiprotease system and immunological mechanisms are the most common etiological factors 2. Recent researches indicated that a noxious environmental insult could evoke the occurrence and development of COPD. Cigarette smoking has been extensively regarded as the most important risk factor for COPD 3, 4. The management and treatment of cigarette smoking-caused COPD is still a huge challenge for all humanity.

Parkinson protein 7 (PARK7)/DJ-1 (DJ-1) was first identified as a gene mutated in a recessively inherited form of early-onset Parkinson disease 5, 6. DJ-1 is a sensitive molecular of oxidative stress with a reactive cysteine at position 106 (Cys106). Under oxidative stress, DJ-1 plays an important role in eliminating hydrogen peroxides, which is oxidized into cysteine sulfnate and sulfonate 7. There is substantial evidence that over-expression of DJ-1 alleviated neurodegeneration caused by oxidative stress in rats 8. Related to its anti-oxidant properties, DJ-1 can not only regulate cell survival and proliferation, but also suppress cell death signaling 9. Additionally, it is reported that DJ-1-deficient mice easily suffered from oxidative stress-induced severe heart failure 10. However, the change of pulmonary DJ-1 was unclear in COPD patients.

Nuclear factor erythroid 2-related factor 2 (Nrf-2) is a key transcription factor that regulates the downstream antioxidant defense system and exerts a significant function in several pulmonary diseases which relates oxidative stress and inflammation 11. Research has found that DJ-1 inhibits Nrf-2 from combining with Kelch-like ECH-associated protein 1 (Keap-1) and attenuates the subsequent ubiquitination of Nrf-2, which is essential for maintaining the stability of the antioxidant enzymes under reactive oxygen species (ROS) stimulation 12, 13. Moreover, Vitamin D and its receptor (VDR) exert a significant role in the metabolism and homeostasis of calcium and phosphate 14. Recently, *in vivo* and *in vitro* experiments indicated that vitamin D supplementation could activate VDR and upregulate Nrf-2 expression in different cells 15-17. However, the association among DJ-1, Nrf-2 and VDR was unclear in COPD patients. The aim of this study was to analyze the associations among DJ-1, Nrf-2 and VDR in lungs of COPD patients based on a case-control study. Our results found that DJ-1 was reduced in serum and lung tissues of COPD patients. Pulmonary VDR and Nrf-2 was decreased in COPD patients. Our data provide evidences that DJ-1 is positively associated with Nrf-2 and VDR in lung tissues of COPD patients.

## Materials and methods

### Reagents

DJ-1, HO-1, NOX-4, Nrf-2 and VDR antibodies were purchased from Cell Signaling Technology (MA, USA). Chemiluminescence (ECL) detection kits were from Advansta (CA, USA). All other chemicals were prepared from Sigma Chemical Co. (MO, USA) unless specifically noted.

### Participants' inclusion

All COPD patients were randomly selected from Anhui COPD Cohort (AHCC) study which was a hospital-based prospective cohort study in Second Affiliated Hospital of Anhui Medical University in China between January 2017 and September 2019. For matched case-control study, patients with COPD were recruited. Pulmonary function was tested in all COPD patients. COPD was confirmed on basis of the American Thoracic Society criteria and the Global Initiative for COPD (GOLD) criteria 1, which forced expiratory volume in 1 s/forced vital capacity ratio was less than 70%. Moreover, all 180 sex-, age- and race-matched control subjects were randomly selected from the physical examination center of Second Affiliated Hospital. Serum was collected between COPD patients and control subjects. In order to evaluate the protein expressions of DJ-1 and oxidative stress markers, 35 human lung tissues of COPD patients were collected in surgery. Moreover, control subjects were paracancerous tissues, which were from histologically confirmed lung cancer patients without COPD. This study was approved by Ethics Committee of Anhui Medical University. All participants have agreed and signed an informed consent.

### Western blotting

Western blotting was performed as the process of previous study 18. Sixty mg lung tissue was added into in the EP tube. Pulmonary protein was extracted using RIPA buffer (500 μL RIPA, 5 μL 1% PMSF, 5 μL phosphatase inhibitor). Lung tissues were homogenized ultrasonic treatment for 15 second on the ice. Lysates were separated through high-speed cryogenic centrifugal. Then, the middle layer was collected. Protein concentrations were detected through BCA protein assay kit. Total 15 μg protein of each sample was added and separated in the 10% SDS-PAGE gel through electrophoresis. Then, protein was transferred to PVDF membranes. Membranes were blocked in no-fat milk for 3 h at room temperature. After, membranes were washed three times using distilled water. Then, primary antibodies including DJ-1 (1:2000), HO-1 (1:2000), NOX-4 (1:2000) were incubated overnight at 4 °C refrigerator, second antibodies of different dilution ratios were continued to incubate 2.5 h at 37 °C incubator. At last, detection was visualized with ECL kit. Each protein was quantified after normalization to band of β-actin using densitometric analysis.

### Immunochemistry (IHC)

Human lung tissues were fixed in formalin and embedded in paraffin. Pulmonary sections (5 μm) were dewaxed and rehydrated according to another research 19. To punch cell membrane and suppress endogenous peroxidase, sections were immersed in PBS containing 0.5% Triton X-100 and 3% H_2_O_2_ for 45 min. Antigen retrieval was performed in boiled citrate solution. Then, the section was blocked in serum, VDR primary antibody (1:200) was incubated at 37 °C incubator for 3.5 h. After washed in PBS three times, conjunction with streptavidin-HRP complex was subsequent incubated for 2.5 h at room temperature. Immunolabelling was evaluated using DAB solution and nuclei were stained with hematoxylin in a dark room. VDR-positive nuclei were calculated in nine randomly selected fields, by two independent pathologists without prior knowledge of the experimental design.

### ELISA

DJ-1 ELISA kits were come from Cusabio, Wuhan, China (https://www.cusabio.com/). Inflammatory cytokines (MCP-1, MIP-1 and TNF-α) were purchased from Wuhan ColorfulGene Biological Technology Co., Ltd. Serum DJ-1 and inflammatory cytokines were measured in a microplate reader using protocols as described previously 20.

### Statistical analysis

Statistical analysis was carried out with SPSS 21.0. All data were expressed as means and medians. Independent sample unpaired t test was evaluated the difference for continuous variables between two groups. Chi-square test was executed for count data between two groups. The correlations among DJ-1, VDR, Nrf-2 and FEV1 were analyzed using Spearman correlation analysis. *P*<0.05 was considered statistically significant.

## Results

### Demographic characteristics and clinical information

Demographic characteristics and clinical information were represented in Table 1. One hundred and eighty COPD patients (73.2% males) and control subjects (63.2% males) were recruited in this project. The median ages were 74.0 and 67.0 years in COPD patients and control subjects, respectively. Routine blood test was carried out. The results indicated that the counts of white blood cells (WBC), neutrophils and monocytes were increased in COPD patients. The number of lymphocytes was increased in COPD patients. Moreover, the levels of inflammatory cytokines in serum were detected in COPD patients and control subjects. We found that CRP, TNF-α, IL-6 and MCP-1 were significantly elevated in COPD patients. Moreover, pulmonary function was measured in COPD patients. The median FVC, FEV1 and FEV1/FVC of current COPD patients was 1.88 L, 40.4% and 51.3%, respectively (Table 1).

### DJ-1 in serum and lung tissues in COPD patients and control cases

**Figure 1:**
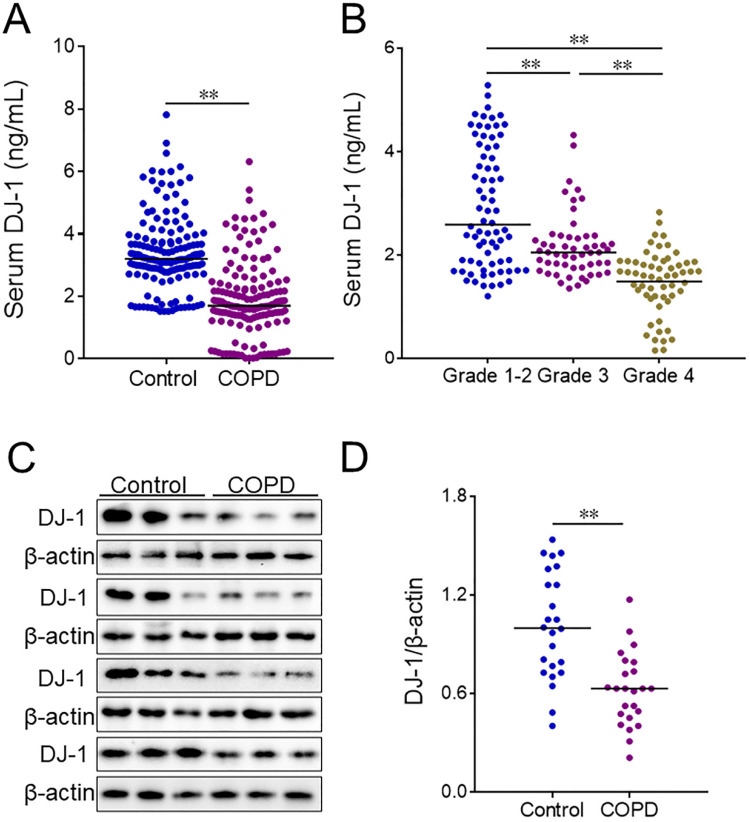
** The level of DJ-1 between COPD patients and control subjects.** Serum and lung tissues were collected from COPD patients and control subjects. The level of DJ-1 was detected in serum using ELISA. (A) The level of serum DJ-1 was detected between COPD patients and control subjects. (B) The level of serum DJ-1 was evaluated in different grades of COPD patients. (C, D) The level of DJ-1 was measured in lung tissues using western blotting. (C) The protein expression of DJ-1 was evaluated in lung tissues between COPD patients and control subjects. (D) Quantitative analysis of scanning densitometry was performed. All data were represented as means ± S.E.M. (N=24). **P<0.01.

Serum DJ-1 was detected using ELISA in COPD patients and control subjects. We found that DJ-1 was reduced in COPD patients (Figure 1A). Moreover, the levels of serum DJ-1 were analyzed in different grades of COPD patients. The results indicated that serum DJ-1 was gradually decreased in parallel with the grades of COPD patients (Figure 1B). In addition, the expression of DJ-1 was detected in lung tissues of COPD patients and control cases through western blotting. Quantitative analysis of scanning densitometry was performed. As shown in Figure 1C and 1D, the expression of pulmonary DJ-1 was obviously down-regulated in COPD patients compared with control subjects.

### Pulmonary markers of oxidative stress in COPD patients and control subjects

**Figure 2:**
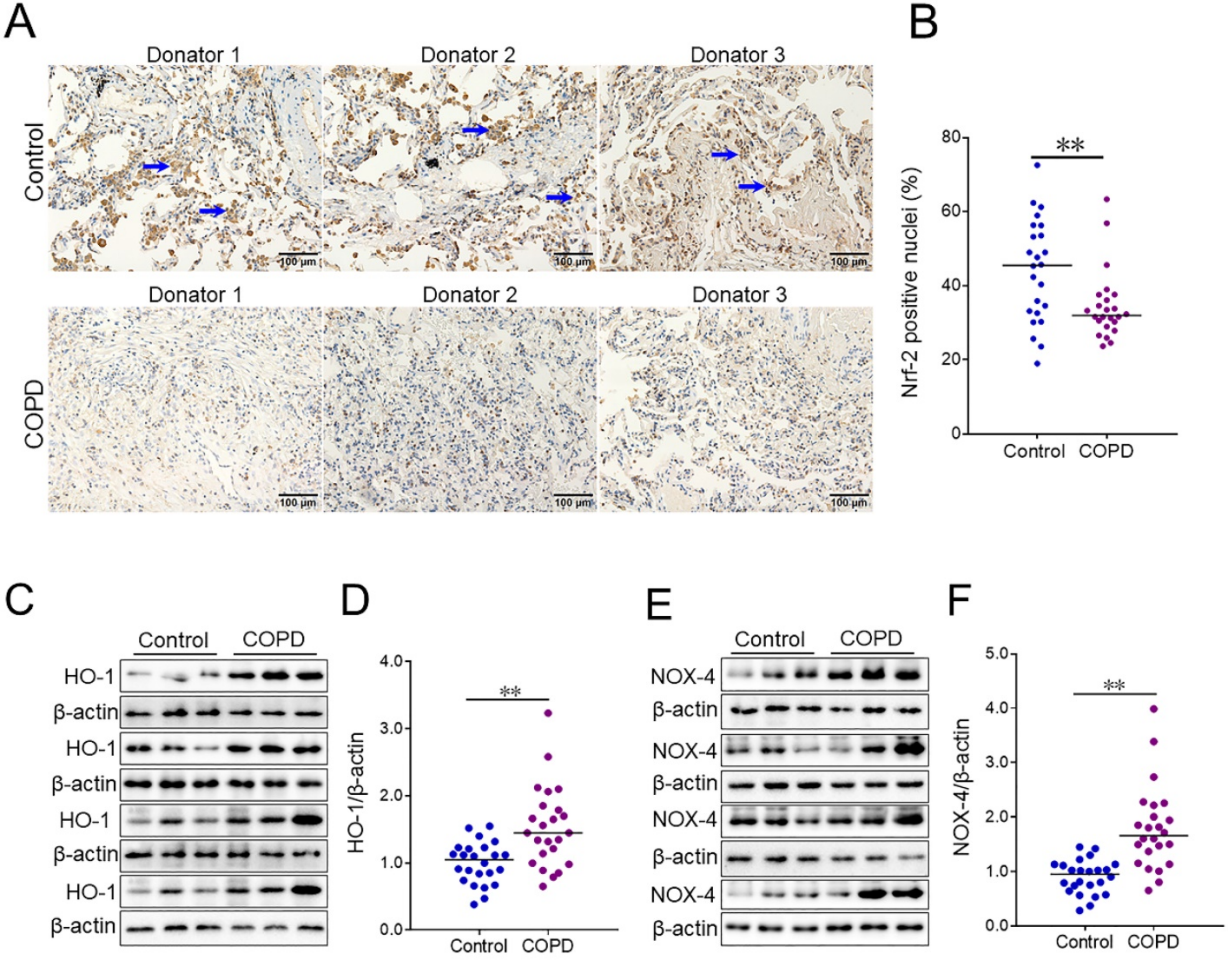
** The levels of oxidative stress markers between COPD patients and control subjects.** (A, B) Pulmonary Nrf-2-positive nuclei were measured using IHC. (A) Representative field. Blue arrows indicate Nrf-2-positive nuclei. Original magnification: ×400. (B) Quantitative analysis of positive nuclei was performed. (C-F) Pulmonary HO-1 and NOX-4 were detected between COPD patients and control subjects through western blotting. (C) Representative bands of HO-1. (E) Representative bands of NOX-4. (D, F) Quantitative analysis of scanning densitometry was performed. All data were represented as means ± S.E.M. (N=24). **P<0.01.

The level of pulmonary Nrf-2 was detected using IHC. The numbers of pulmonary Nrf-2-positive nuclei were compared in COPD patients and control subjects. As shown in Figure 2A and 2B, the number of pulmonary Nrf-2-positive nuclei was decreased in COPD patients compared with control subjects. Additionally, the markers of oxidative stress were measured in lung tissues of COPD patients and control subjects. As shown in Figure 2C-2F, the expression of pulmonary heme oxygenase-1 (HO-1) and NADPH oxidase 4 (NOX-4) was increased in COPD patients.

### Pulmonary VDR-positive nuclei in COPD patients and control subjects

**Figure 3:**
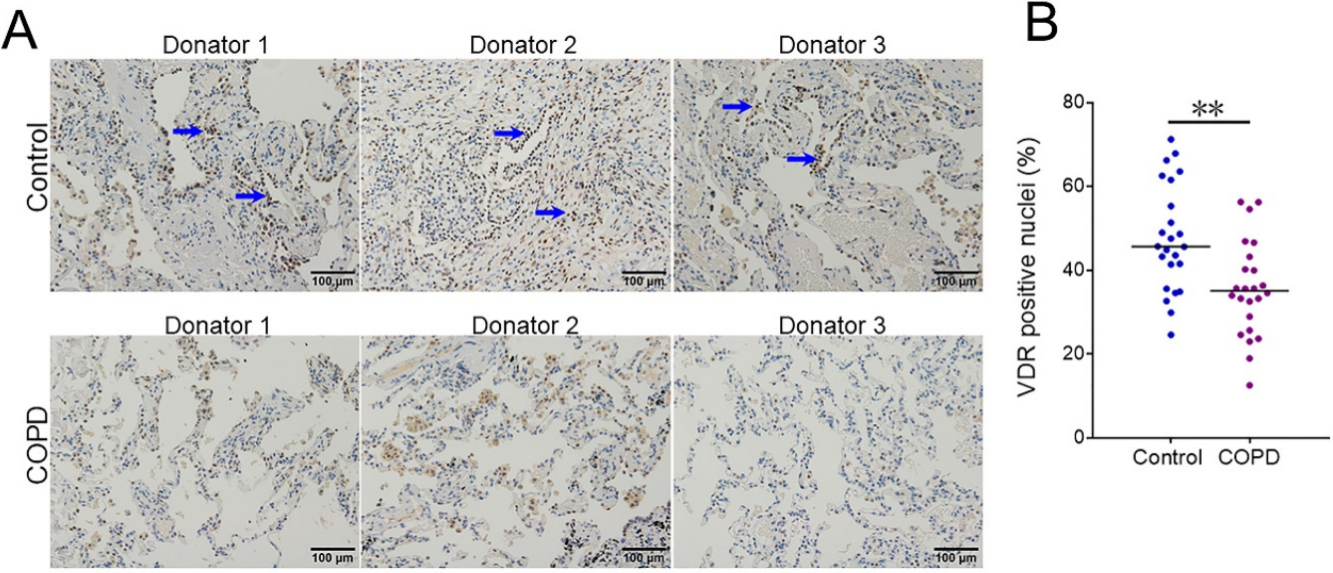
** The level of pulmonary VDR-positive nuclei between COPD patients and control subjects.** (A) Pulmonary VDR-positive nuclei were measured using IHC. Blue arrows indicate VDR-γ-positive nuclei. Original magnification: ×400. (B) Quantitative analysis of positive nuclei was performed. All data were represented as means ± S.E.M. (N=24). **P<0.01.

VDR-positive nuclei were analyzed in lung tissues between COPD patients and control subjects using IHC. As shown in Figure 3A and 3B, the number of pulmonary VDR-positive nuclei was less in COPD patients than these in control subjects.

### Correlations of pulmonary function with pulmonary DJ-1, Nrf-2 and VDR in COPD patients

**Figure 4:**
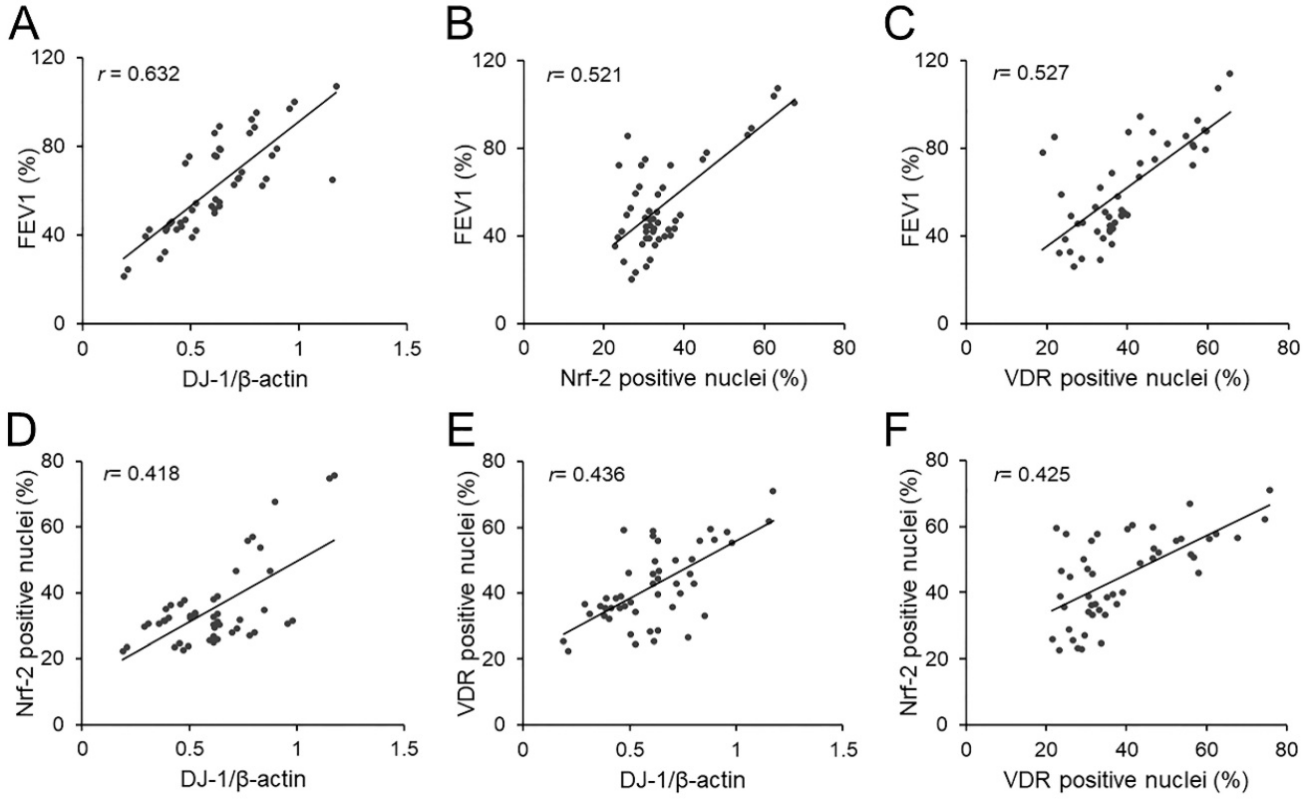
** Associations among FEV1, DJ-1, Nrf-2 and VDR in COPD patients.** (A-C) Associations of FEV1 and pulmonary DJ-1, VDR and Nrf-2 were analyzed in COPD patients. (A) FEV1 vs DJ-1. (B) FEV1 vs Nrf-2. (C) FEV1 vs VDR. (D-F) Associations among DJ-1, VDR and Nrf-2 in lung tissues of COPD patients. (D) Nrf-2 vs DJ-1. (E) VDR vs DJ-1. (F) VDR vs Nrf-2.

The correlations of FEV1 with pulmonary DJ-1, Nrf-2 and VDR were analyzed. As expected, there was an obviously positive correlation between FEV1 and pulmonary DJ-1 expression in COPD patients (*r*=0.632, *P*<0.01) (Figure 4A). Moreover, we found that pulmonary DJ-1 expression was significantly associated with Nrf-2- and VDR-positive nuclei in lung tissues of COPD patients (*r*=0.521, *P*<0.01; *r*=0.527, *P*<0.01) (Figure 4B and 4C). In addition, the associations of DJ-1 with Nrf-2 and VDR were evaluated in lung tissues of COPD patients. As shown in Figure 4D and 4E, DJ-1 expression was notably correlated with Nrf-2- and VDR-positive nuclei in lung tissues of COPD patients (*r*=0.418, *P*<0.05; *r*=0.436, *P*<0.01). Moreover, the association between VDR-positive nuclei and Nrf-2-positive nuclei was analyzed. As shown in Figure 4F, there was an obvious positive correlation between VDR-positive nuclei and Nrf-2-positive nuclei in lung tissues of COPD patients (*r*=0.425, *P*<0.05).

## Discussion

In the current research, we mainly detected the levels of DJ-1, VDR, Nrf-2 and related markers of oxidative stress between COPD patients and control subjects. The associations of FEV1 and DJ-1, VDR and Nrf-2 were evaluated in COPD patients. The present research mostly found that: (1) Pulmonary and serum DJ-1 was decreased in COPD patients; (2) Pulmonary VDR and Nrf-2 were reduced in COPD patients; (3) Pulmonary HO-1 and NOX-4 were elevated in COPD patients; (4) There were positive correlations among pulmonary function, DJ-1, VDR and Nrf-2 in lung tissues of COPD patients.

The pathogenesis and pathology of COPD are complex, but excessive oxidative stress is an important cause and mechanism of COPD 2. Compelling data suggest that oxidative stress derived from ROS present in COPD patients and evoked several inflammatory and immune stimuli in epithelial cells of the airways 21. Recently, a research found that the markers of oxidative stress were elevated in peripheral blood mononuclear cells derived from COPD patients 22. To our knowledge, Nrf-2 is a key transcription factor that regulates the downstream antioxidant defense system and exerts a significant function in COPD 23. The previous *in vivo* experiment found that cigarette smoking, the primary cause of COPD, evoked ROS excessive production and Nrf-2 downregulation in the human lung alveolar epithelium cell line 24. Meanwhile, our results indicated HO-1 and NOX-4 were increased in lungs of COPD patients. On the contrary, pulmonary Nrf-2-positive nuclei was reduced in COPD patients.

It is commonly understood that HO-1 is the important downstream molecule of Nrf-2. However, NOX-4 have been proved to be the main source of ROS under various pathological conditions. Cigarette smoking is the most important risk factor for COPD 3, 4. Mounting evidences proved that cigarette smoking induced the production of ROS and evoked oxidative stress 25. Simultaneously, cigarette smoking elevated the expression and activity of HO-1 in mouse cerebral vascular endothelial cells 26. When human bodies suffer from ROS attack, the bodies may activate the anti-oxidization system in the body to eliminate ROS. It is quite possible that these two molecules of opposite functions showed similar expression in human bodies. HO-1 is mainly induced under several stimuli such as hypoxia, oxidative stress, cytokines, cigarette smoke and heavy metals in biological systems 27. Different intracellular signaling molecules and transcription factors are associated with HO-1 expressions such as Nrf-2, Tramtrack and Broad complex (BTB), activator protein-1 (AP-1), nuclear factor kappa-B (NF-κB) and Mitogen-activated protein kinase (MAPK) 28. Though cigarette smoking inhibited Nrf-2 nuclear translocation, it also activated several inflammatory signaling, such as AP-1, NF-κB and MAPKs, and then induced oxidative stress 29, 30. An earlier study found that pulmonary Nrf-2 was reduced in COPD patients 31. However, recent researches indicated HO-1 was increased in COPD patients 32, 33. The human body is an organic and complex whole. Intracellular redox imbalance results in different trends of Nrf-2 and HO-1 in human bodies. Maybe those reasons partially explain why Nrf-2 is decreased, HO-1 and NOX-4 are increased in COPD patients.

DJ-1 is a redox sensitive molecular and stabilizer of Nrf-2 10. Several researches indicated that DJ-1 exerted important roles in the defense against oxidative stress. The data reveal that DJ-1 inhibited Nrf2 from combining with Keap-1 and attenuated the subsequent ubiquitination of Nrf-2, which was essential for maintaining the stability of the antioxidant enzymes under ROS stimulation 12, 13. In the absence of oxidative stress, DJ-1-defcient mice showed more severe myocardial injury in response to ischemia 34. In the Parkinson's disease, oxidative stress mediated reduction in DJ-1 enhances CML α-synuclein in cells 35. Simultaneously, the level of DJ-1 was detected in serum and lung tissues. Our results suggested that the level of DJ-1 was decreased in serum and lung tissues of COPD patients. Moreover, the levels of DJ-1 were further analyzed in different grades of COPD patients. The results found that serum DJ-1 was gradually decreased in parallel with pulmonary function decline in COPD patients. Not only that, correlation analysis indicated that the expression of DJ-1 was positively associated with Nrf-2-positive nuclei in lung tissues of COPD patients. These results provide evidences that DJ-1 reduction is correlated with the downregulation of Nrf-2 in lung tissues of COPD patients.

VDR was the nuclear receptor for the biologically most active vitamin D metabolite 1α,25-dihydroxyvitamin D3, which not only exerted a significant role in the metabolism and homeostasis of calcium and phosphate, but also involved in the process of cellular proliferation and differentiation, anti-inflammatory and anti-oxidative function 14, 35-38. Increasing evidences indicated that VDR played an important function in the process of COPD during the past few decades 39. Recently, *in vivo* and *in vitro* experiments indicated that vitamin D supplementation could activate VDR and upregulate Nrf-2 expression in different types cells 15-17. Vitamin D deficiency in obese rats exacerbated nonalcoholic fatty liver disease and elevated oxidative stress marker in liver 40. This data suggested Nrf-2 may be the downstream protein of VDR. In the current study, we found that VDR- and Nrf-2-positive nuclei were decreased in lung tissues of COPD patients. In addition, we found that VDR was positively associated with DJ-1 and Nrf-2 in lung tissues of COPD patients. These results indicated DJ-1 reduction and VDR downregulation may together contribute to pulmonary Nrf-2 degression. Pulmonary Nrf-2 downregulation further resulted in the insufficiency of antioxidant defense system, damaged pulmonary epithelial cells and finally evoked the occurrence and development of COPD. These results provide a possible mechanistic explanation by which the downregulation of DJ-1 and VDR cause pulmonary function decline in COPD patients.

There are a few defects in the current study. Firstly, this was only a case-control study, so the causal relationship between pulmonary Nrf-2 reduction and COPD was unclear, further animal experiments *in vivo* would help resolve this confusion. Secondly, whether DJ-1 and VDR downregulation associating with Nrf-2 reduction involved in COPD needs to further confirm *in vitro* and *in vivo* experimental studies. Thirdly, the mechanism of reduction of Nrf-2 and elevation of Nrf-2' downstream target protein were obscure in COPD patients. *In vivo* and *in vitro* experiments may demonstrate the mechanism. Fourthly, this was a single-center and small sample size study, a larger sample size and multicenter study is required in the future research.

## Conclusions

In summary, this study mainly analyzed the correlations among pulmonary function, DJ-1, VDR and Nrf-2 in COPD patients in a hospital-based case-control study. Our results demonstrate that pulmonary and serum DJ-1 is decreased in COPD patients. Meanwhile, pulmonary VDR and Nrf-2 are reduced in COPD patients. Correlation analysis suggested that pulmonary DJ-1, VDR and Nrf-2 is positively associated with FEV1 in COPD patients. In addition, there are positive correlations among DJ-1, VDR and Nrf-2 in lung tissues of COPD patients.

## Figures and Tables

**Table 1:** Demographic and biochemical characteristics between COPD patients and control subjects

Variables	COPD (n=180)	Control (n=180)	*P*
Age (years)	74.0 (68.5, 82.0)	67.0 (50.3, 79.6)	0.437
Male, n (%)	132 (73.2)	114 (63.2)	0.351
Hospital stay (day)	10.0 (7.0, 14.0)	N.S.	N.S.
WBC (10^9^/L)	6.79 (5.07, 9.52)	5.32 (4.32, 6.93)	<0.01
Neutrophils (10^9^/L)	4.74 (3.38, 7.22)	3.15 (2.21, 3.96)	<0.01
Lymphocyte (10^9^/L)	1.08 (0.78, 1.45)	2.15 (1.72, 2.63)	<0.01
Monocyte (10^9^/L)	0.47 (0.33, 0.68)	0.35 (0.29, 0.44)	<0.05
CRP (μg/mL)	88.3±9.6	36.5±7.4	<0.01
TNF-α (ng/mL)	103.5±7.9	30.3±6.2	<0.01
IL-6 (pg/mL)	48.6±10.6	19.6±7.6	<0.05
MCP-1 (pg/mL)	206.5±42.5	58.6±7.6	<0.01
FVC (L)	1.88 (1.36, 2.47)	N.S.	N.S.
FEV1 (%)	40.4 (27.6, 64.8)	N.S.	N.S.
FEV1/FVC (%)	51.3 (43.3, 58.5)	N.S.	N.S.
